# The Promising Ability of *Humulus lupulus* L. *Iso*-α-acids *vs*. Diabetes, Inflammation, and Metabolic Syndrome: A Systematic Review

**DOI:** 10.3390/molecules26040954

**Published:** 2021-02-11

**Authors:** Maria Ponticelli, Daniela Russo, Immacolata Faraone, Chiara Sinisgalli, Fabiana Labanca, Ludovica Lela, Luigi Milella

**Affiliations:** 1 Department of Science, University of Basilicata, Viale dell’Ateneo Lucano 10, 85100 Potenza, Italy; daniela.russo@unibas.it (D.R.); immacolata.faraone@unibas.it (I.F.); chiara.sinisgalli@unibas.it (C.S.); fabiana.labanca@unibas.it (F.L.); ludovica.lela@unibas.it (L.L.); 2 Spinoff BioActiPlant s.r.l., Viale dell’Ateneo Lucano 10, 85100 Potenza, Italy

**Keywords:** *Humulus lupulus* L., *iso*-α-acids, reduced *iso*-α-acids, metabolic syndrome, PPAR receptors

## Abstract

For centuries, natural medicines have represented the only option for treating human diseases and, nowadays, plant phytochemicals are considered as promising compounds to treat or prevent chronic conditions. Among them, hop flowers (*Humulus lupulus* L.), typically used in brewing industries to give the typical aroma and flavor to beer, have attracted particular attention for their health promoting properties. Several *in vivo/vitro* studies and human interventional trials have demonstrated the beneficial effects of these molecules on weight gain, lipid metabolism, glucose homeostasis, insulin sensitivities, and inflammation by acting on different targets. All these activities suggest a possible role of bitter hop acid in preventing metabolic syndrome and its related diseases. A systematic quest on PubMed and Scopus databases was performed to identify pre-clinical and clinical studies focusing on this topic. This systematic review summarizes the results obtained by different cell lines, animal models, and human interventional trials to propose *iso*-α-acids as medical nutrition therapy to treat or prevent metabolic syndrome and its related disorders as diabetes, dislipidemia inflammation, etc.

## 1. Introduction

Metabolic Syndrome (MetS) is a condition characterized by a complex mosaic of metabolic abnormalities, including visceral obesity, impaired glucose tolerance, insulin resistance leading to type II diabetes, dyslipidemia, and atherosclerotic cardiovascular diseases. In the pathogenesis of MetS either acquired or genetic factors are involved, leading to the rise of oxidative stress and a systemic inflammatory process. Indeed, systemic inflammation is a constant feature of metabolic syndrome since obesity is generally associated with low-grade inflammation of white adipose tissue resulting in the chronic activation of the innate immune system. The excess in macronutrients results in a dysregulated secretion of pro-inflammatory adipokines and cytokines from adipose tissue such as leptin, interleukin-6 (IL-6), tumor necrosis factor-α (TNF-α), monocyte chemoattractant protein-1 (MCP-1), and resistin [1]. These inflammatory mediators are not only limited to adipose tissue but also reach the liver through the portal vein, and finally, other peripheral tissues, like vascular tissues, where they can induce atherosclerosis, hypertension, and vascular insulin resistance [2]. According to the National Cholesterol Treatment Program Adult Treatment Panel III (NCEP–ATP III), it is possible to diagnose MetS when three or more of the following criteria fulfilled: waist circumference over 35 inches for women or 40 inches for men, fasting triglycerides level over 150 mg/dL, fasting HDL-C level less than 50 mg/dL for women and 40 mg/dL for men, blood pressure over 130/85 mmHg, and fasting blood sugar over 100 mg/dL [3].

Due to the worldwide increase in obesity, MetS represent a serious public health problem; thus, researchers have moved their attention to the study of this condition. Generally, the common treatment of metabolic disorders consists of drastic dietary interventions based on the replacement of refined carbohydrates with proteins and fibers, the reduction of saturated fats, and the increment of poly-unsaturated oils. Diets are often associated with pharmacologic therapies leading to the decrease of obesity incidence, the treatment of diabetes, and the reduction of heart disease by decreasing LDL cholesterol and reducing high blood pressure [4]. However, dietary interventions are difficult to implement in obese patients, and different drugs have shown low efficacy and a high number of adverse reactions. For these reasons, new safe and effective therapeutic strategies are demanded. In particular, several studies have described the beneficial effects of various natural extracts and active principles and their potential role in the improvement of MetS related diseases, as diabetes, dislipidemia, oxidative stress, etc. [5,6,7,8]. Moreover an increasing attention has been paid to bitter compounds able to exert their activity interacting with specific gastrointestinal receptors [9]. To the best of our knowledge, the effectiveness of bitter compound present in *Humulus lupulus* L. using a systematic approach have not been reported yet. Hop (*Humulus lupulus* L.) is globally known since it is the brewing industry’s raw material. Hop’s female inflorescences, also called hop cones, contain resin glands capable of secreting a yellow resinous powder known as lupulin. It is rich in different active compounds that contribute to beer aroma and stability, and that can be classified as volatile oils, bitter acids, and polyphenols [10]. Several of these molecules have been demonstrated to exert different biological activities. In particular, during the last years, hop bitter acids have been demonstrated to be possible candidates for treating and/or preventing several human disorders, including cancer, diabetes mellitus, and cardiovascular diseases [11,12]. Due to the described effects, our attention was focused on metabolic syndrome-related diseases. Several studies have recently described the ability of *iso*-α-acid and reduced *iso*-α-acid to improve metabolic disorders by affecting body weight, glucose tolerance, insulin resistance, lipid metabolism, and inflammatory mediators.

This review analyzes, using a systematic approach, research papers available to date to make a comprehensive knowledge of the recent achievements in the field of hop bitter acid activity in preventing and treating obesity, hyperlipidemia, insulin resistance, systemic inflammation, and atherosclerosis, starting from the bioavailability and metabolism of these compounds.

## 2. Bitter Hop acid Chemistry

**Figure 1:**
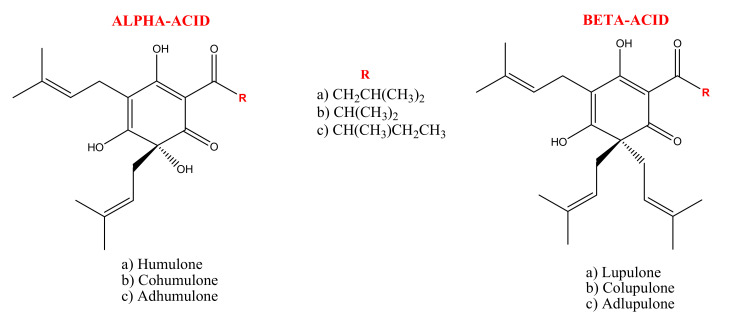
Representation of α-acids or humulones and β-acids or lupulones.

Hop bitter acids (25% or even more of the dry weight of hop) are phloroglucinol derivatives consisting of two series of compounds: α-acids or humulones and β-acids or lupulones (Figure 1). These two groups comprise three compounds whose chemical structure differs in the side chains’ composition. In fact, the α-acids’ side chain may be derived from hydrophobic amino acids, leucine, valine, and isoleucine, for humulone/lupulone, cohumulone/colupulone, and adhumulone/adlupulone, respectively [13]. The amount of α-acids depends strongly on the hop variety and it reflects the quality of the raw material used in brewer industries. During beer production, α-acids are susceptible to an isomerization process, via an acyloin-type ring contraction, by boiling hops or hop extract in aqueous wort medium at high pH. As a result, the most hydrophilic and bitter *iso*-α-acids are produced. Depending on the spatial arrangement of the tertiary alcohol function at C(4) and the prenyl side chain at C(5) it is possible to distinguish two diastereomeric pairs of *iso*-α-acids: cis-*iso*-α-acids and trans-*iso*-α-acids. It is also possible to obtain these compounds in laboratory practices by boiling α-acids in the presence of a catalyst as divalent cations, in alkaline media or by irradiation, with UV light of an α-acids methanolic solution (photoisomerization acts in a stereoselective ways and exclusively forms trans-isomers) [14,15].

**Figure 2:**
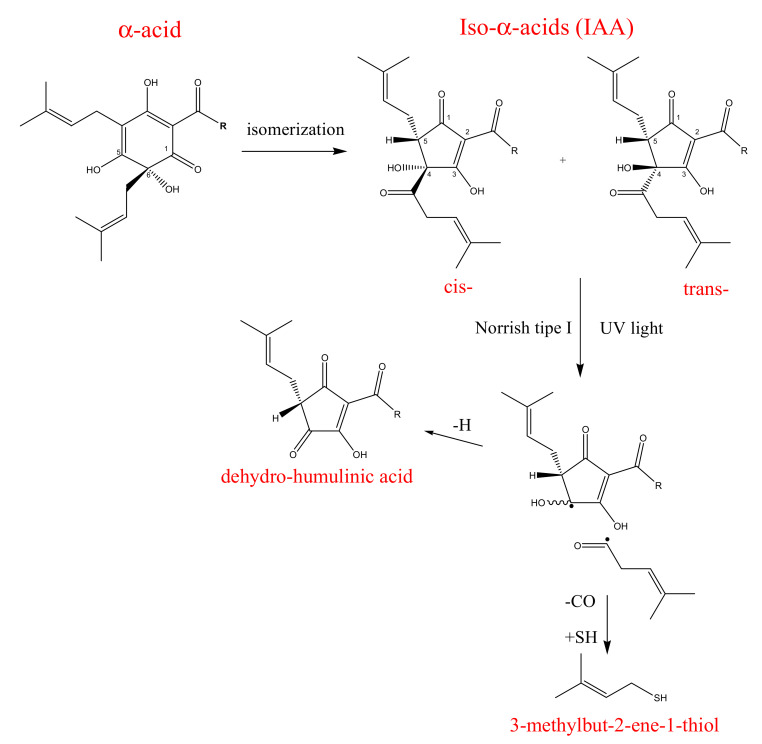
Isomerization of α-acids and degradation of *Iso*- α-acids by Norrish Type I reaction.

With the isomerization process from the three main α-acids analogs, six stereoisomers of *iso*-α-acids are produced: *cis*-isohumulone and *trans*-isohumulone, *cis*-isocohumulone and *trans*-isocohumulone, *cis*-isoadhumulone and *trans*-isoadhumulone. The ratio between the two isomers is 68:32 in favor of the *cis* isomers, which are more stable (half-life ≈ 5 years) than trans-isomers (half-life ≈ 1 year) due to the lower steric impedance which exists between the two large vicinal side chains. However, both isomers in the presence of sunlight and oxygen are prone to the Norrish Type I reaction with the final generation of the so-called skunky thiol (3-methylbut-2-ene-1-thiol), which, together with dehydro-humulonic acid, is responsible for beer decomposition [13] (Figure 2). For this reason, beer is historically bottled in brown or green, light-proof glass. Recently, several light-stable derivatives, the reduced *iso*-α-acids, have been chemically synthesized from natural *iso*-α-acids. In fact, by reducing the weak double bonds or carbonyl group in the side chain of the *iso*-α-acids into the stronger single bonds, it is possible to prevent the photolytic cleavage, which produces the unwanted skunky thiol [16].

**Figure 3:**
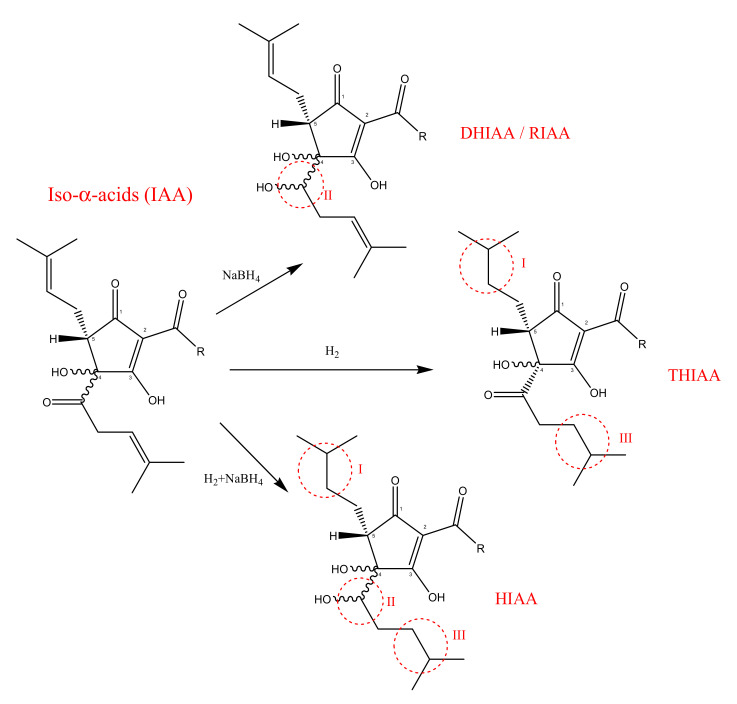
Reduction of *iso*-α-acids: I, III sites of increased hydrophobicity; II, III sites of photolytic cleavage prevention.

Depending on the number of the hydrogen atoms incorporated during the reduction, it is possible to distinguish three different reduced *iso*-α-acids (Figure 3):

- Dihidro-*iso*-α-acids (DHIAA) also known as Rho-*iso*-α-acids (RIAA): obtained from the sodium borohydride reduction of the carbonyl group in the side chain at C(4);
- Tetrahydro-*iso*-α-acids (THIAA): obtained by the hydrogenation of both *iso*-α-acids double bonds in the side chain;
- Hexahydro-*iso*-α-acids (HIAA): are obtained by a combination of two reactions: the reduction of the carbonyl group and the hydrogenation of the double bonds in the *iso*-α-acids.

These reduced compounds are light stable and preserve the beer’s bitter flavor for a longer time. Additionally, tetrahydro- and hexahydro-*iso-*α-acids can stabilize beer foam, thanks to an increase in their hydrophobicity. However, as previously mentioned, *iso*-α-acids and their reduced derivatives are useful not only for industrial purposes but also for their potential application in medical areas. Thus, this review resumes a systematic analysis of the studies which have described the potential role of bitter hop compound in improving metabolic syndrome diseases.

## 3. Results and Discussion

### 3.1. Study Characteristics

**Figure 4:**
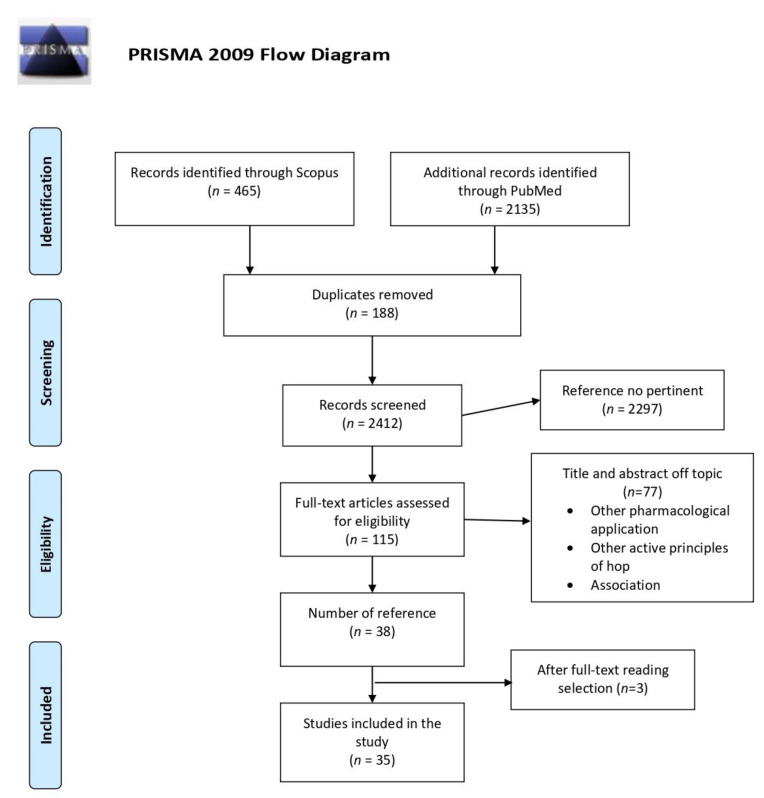
Diagram of systematic review literature search results based on the Preferred Reporting Items for Systematic Reviews and Meta-Analyses (PRISMA) statement.

This systematic review has followed the recommendation of the PRISMA statement [17] (Figure 4), including articles published between 2000 and 2020 using PubMed (http://www.ncbi.nlm.nih.gov/pubmed (accessed on 11 February 2021)) and Scopus (http://www.scopus.com (accessed on 11 February 2021)) as databases including all published reports until December 2020. The text keywords used are: hop, *Humulus lupulus*, bitter acid, *iso*-humulone, and *iso*-α-acid, paired with the following words: metabolic syndrome, obesity, inflammation, diabetes, pharmacokinetics, clinical trial. Research works were limited to English-language publications.

**Figure 5:**
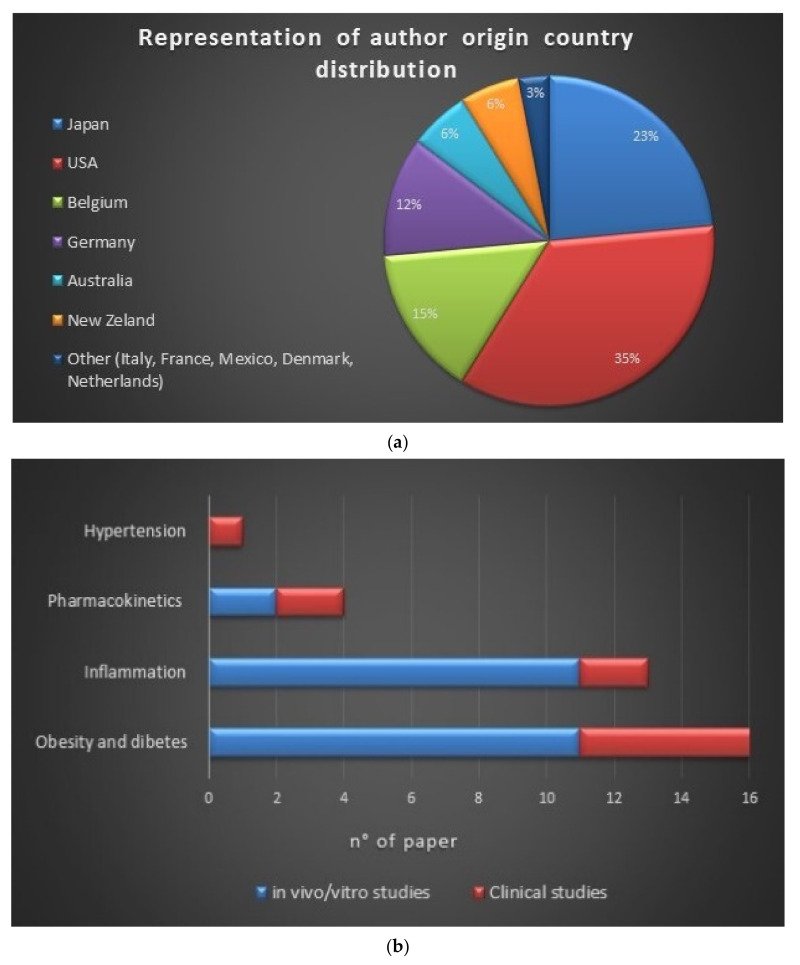

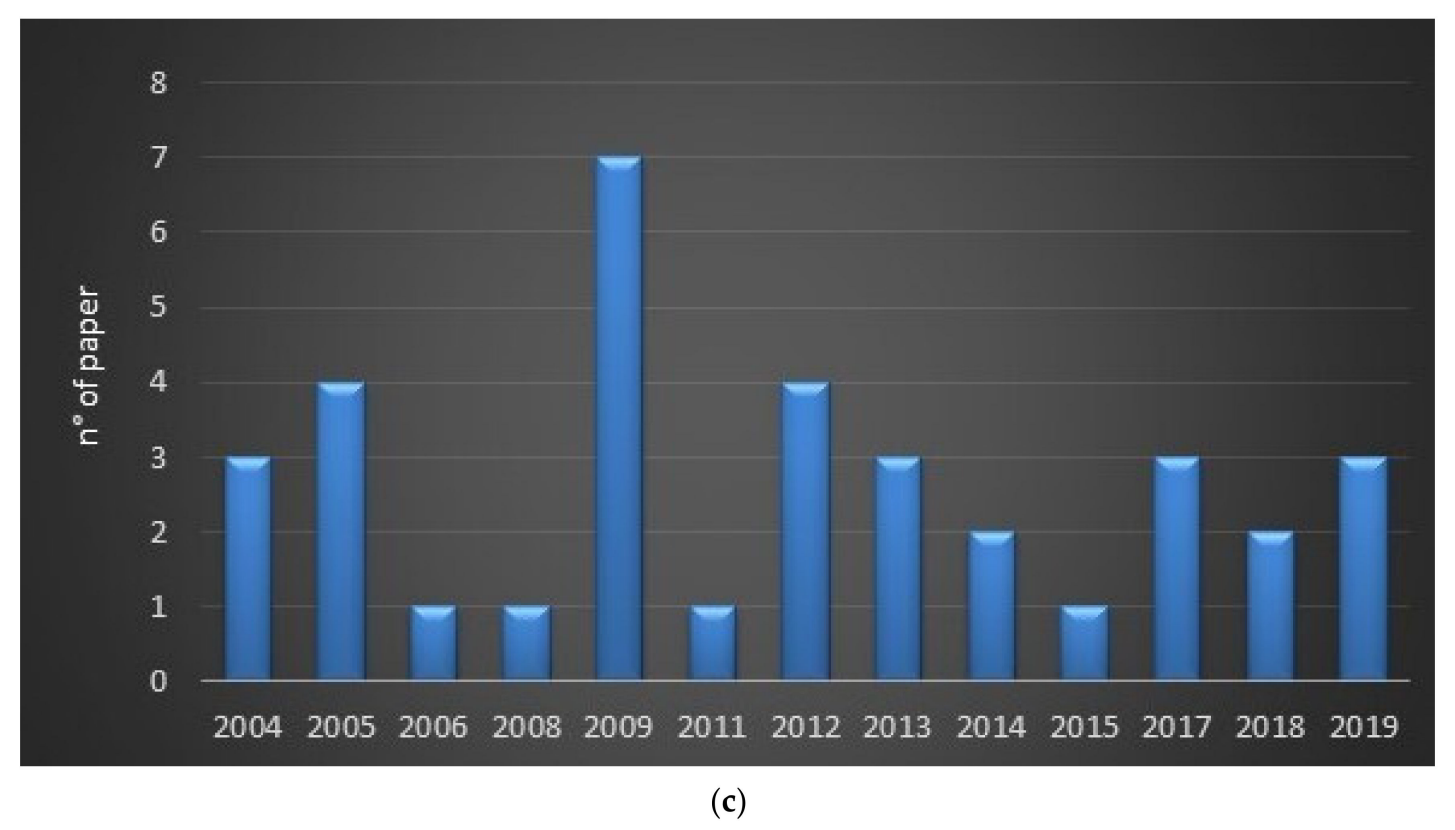
(**a**) Schematic representation of author origin country distribution, (**b**) number of articles per argument (blue indicates the number of pre-clinical studies, red indicates clinical studies), (**c**) distribution of the selected studies by year of publication.

The initial selection provided 2600 articles, of which 2135 were found on PubMed and 465 on Scopus. Among the 2600 items, only 303 were related to the research subject; from these 303 documents, 188 were duplicates. Out of those 115 studies, 77 were off-topic basing on the exclusion criteria, while 3 did not contain experimental data congruent with the topic. The final reference list comprises 35 items from 11 countries [USA 35%, Japan 23%, Belgium 15%, Germany 12%, Australia 6%, New Zeland 6%, Other (Italy, France, Mexico, Denmark, and the Netherlands) 3%)] (Figure 5a).

**Figure 6:**
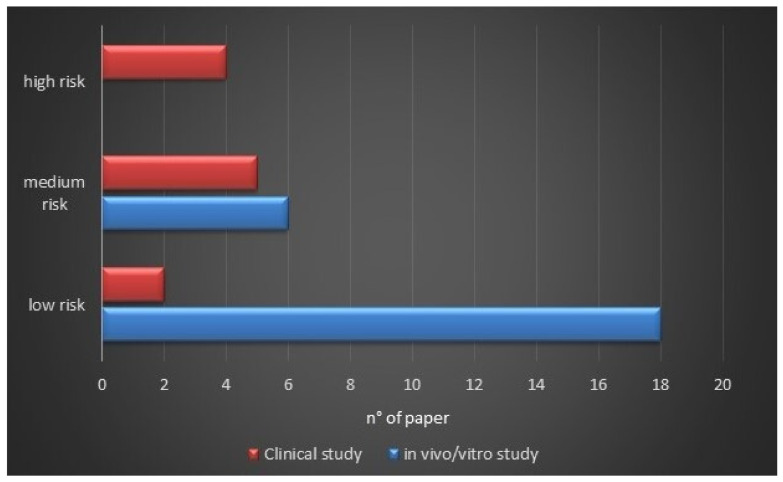
The quality assessment is based on a checklist adapted from the Cochrane Handbook for Systematic Reviews of Interventions. The pre-clinical (indicated by blue bar) and clinical studies (indicated by red bar) have been classified in high, medium, and low risk of bias.

This systematic review included 24 in vitro/vivo reports and 11 clinical investigations (Figure 5b). In vitro studies included colorimetric and enzymatic assays, cytometric analyses, immunofluorescence techniques, and western blots. *In vivo* experiments were carried out on animal models like mice, rats, and rabbits to determine the effect of *iso*-α-acid and reduced *iso*-α-acid on body weight, glucose tolerance, insulin resistance, lipid metabolism, and inflammation. Clinical investigations were performed on diabetic, pre-diabetic, or healthy volunteers to evaluate the effect of *iso*-α-acid and reduced *iso*-α-acid on obesity, diabetes, and inflammation. For *in vivo* and clinical investigations, dosages, administration routes, frequency of administration, and the duration of the therapy have been cited in all cases. Risks bias assessment, based on a checklist adapted from the Cochrane Handbook for Systematic Reviews of Interventions, is reported in Figure 6. The low number of clinical trials with a low risk of bias is due to the lack of trials, including a large number of subjects.

The results obtained, in the various studies on *iso*-α-acids’ metabolic syndrome effects, have been summarized as follows.

### 3.2. Pharmacokinetics of Hop iso-α-acids

Usually, hop-derived bitter acids contents in beer can vary from 10 up to 100 mg/L, while commercially available hop-based food supplements can contain up to 400 mg of hop bitter acid. This high concentration of bitter hop acids may explain the healthy activities of *iso*-α-acids and reduced *iso*-α-acids [17]. Thus, researchers have set the goal of studying the absorption, distribution, metabolism, and excretion (ADME) process of hop *iso*-α-acids (IAA) through *in vitro/vivo* investigation and on healthy human volunteers after beer consumption.

One of the most important pharmacokinetic properties of drugs is their oral bioavailability, which is known to be affected by different intestinal factors such as permeation, efflux, intestinal metabolism, and colon macrobiotic metabolism. It has been shown that to improve intestinal permeability, molecules must have a partition coefficient value (logP) of 2–3, and net charge in the pH range of 5.5–7.0 [18]; moreover, to spread through cell membranes, molecules must have a length not exceeding the threshold of about 1.5 nm [19]. Hop isomerized acids and their reduced derivatives have not only a molecular length (Table 1) of less than 1.5 nm but also a good grade of lipophilicity, indicating a great tendency to be transported toward cell membranes [19].

Considering this evidence, to investigate epithelial transport, in both absorptive and secretive direction, IAA and their reduced derivatives (DHIAA and THIAA) were in vitro tested on human Caco-2 cell monolayers in different concentration (30, 60, and 120 μM). From this investigation, it was seen that hop derivatives are subjected to a passive diffusion mechanism in both absorptive and secretive direction, as indicated by the lack of saturation effects and by a linear dose- and time-transport relationship. Further, a comparison of the membrane permeability of the three compounds showed that IAA and THIAA had a higher membrane permeability in both absorptive (apical P_app_ range 1.6–5.6 × 10^−6^ cm/s) and secretive (basolateral P_app_ range 5.7–16.3 × 10^−6^ cm/s) direction than DHIAA. One of the limiting factors of DHIAA transport could include phase-II metabolism. In fact, through the enzymatic hydrolysis of the cellular fraction containing the three samples, it was demonstrated that the cellular fraction of DHIAA underwent conjugation reactions as glucuronide or sulphate in an amount up to 60% [17]. In another in vitro study, phase-I metabolism was also investigated as a possible factor of a reduced bioavailability of hop isomerized acid. It was found that contrarily to DHIAA, the bioavailability of IAA may be affected by phase-I metabolism, as demonstrated in rabbits’ liver microsomes incubated with IAA (20 μM for 120 min incubation). In particular, the 3-methyl-2-butenyl side chains and the isohexenoyl side chain of IAA were susceptible to oxidation, either at the double bonds or in the allylic positions. Thus, IAA’s microsomal metabolism resulted in the formation of *trans*-humulinic acids and *cis*-humulinic acids, in addition to *trans* and *cis* pairs of hydroperoxy-alloiso-α-acids and hydroxylated alloiso-α-acids. It is also possible to observe IAA’s degradation into cyclic substances such as tricyclohumols [20]. Additionally, through the use of in silico metabolism pathway prediction modelling software such as “MetaPred”, it was suggested that the cytochrome P450 2C9 was primarily responsible for IAA oxidation [21].

These results have been confirmed by *in vivo* studies conducted on New Zealand white rabbits. In this study, 25 mg/kg of IAA, DHIAA, and THIAA (corresponding with a dose of around 500 mg for a human weight of 60–70 kg) were orally or intravenously administered to evaluate their bioavailability. It was found that the absolute orally or intravenous bioavailability for IAA (13%) was lower than the bioavailability of DHIAA and THIAA (28% and 23%, respectively), confirming the trend of IAA to be metabolized by phase-I metabolism. Further, samples of feces and urine were subjected to enzymatic hydrolysis with a mixture of sulfatase and glucuronidase activities to evaluate possible phase-II-metabolism of IAA, DHIAA, and THIAA. It was seen that up to 22% of the excreted amount of DHIAA was conjugated as sulphate or glucuronide, while no significant conjugation in urine or feces was showed for IAA or THIAA. This confirms the reduced bioavailability of DHIAA by phase-II-metabolism previously demonstrated *in vitro* [17].

As regards the pharmacokinetic values of IAA and their reduced derivatives, they were evaluated on healthy volunteers. Through the analysis of blood samples, it was established that, after beer consumption, the reduced IAA showed a longer half-life (t_1/2_~45–46 min for DHIAA and THIAA, respectively) than natural IAA (t_1/2_~30 min) [16,22]. Only unmodified DHIAA was detected in the urine after consumption, while no THIAA [16] and only trace amounts of unmodified IAA [22] were excreted through urine. These results confirm previous data obtained on the rabbit model since DHIAA and THIAA were excreted in the amount of 12% and 1%, respectively [17]. Furthermore, enzymatic hydrolysis of plasma and urine samples confirm the possible phase I metabolism for IAA and THIAA and a phase II metabolism for DHIAA as the most likely limiting factor of bioavailability [16].

### 3.3. Pre-clinical Investigation

#### 3.3.1. Hop Isomerized Compounds Activity on PPARs

Pharmacological treatments for MetS condition aim to regulate nuclear transcription factors named Peroxisome proliferator-activated receptors (PPARs), which play an essential role in regulating storage and catabolism of dietary fats and glucose, cholesterol metabolism, and in adipocyte differentiation [23,24].

**Figure 7:**
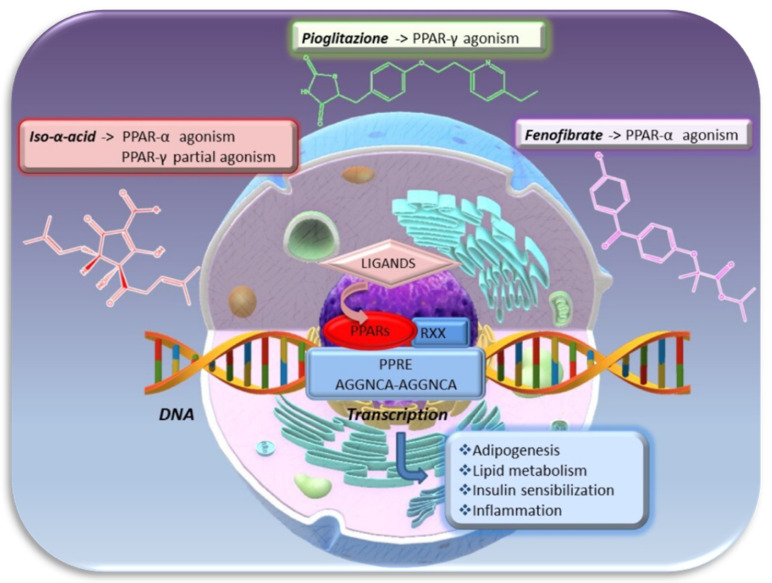
Transcriptional activation mediated by peroxisome proliferator-activated receptors (PPARs). PPARs form a heterodimer with the retinoid-X receptor (RXR) and, as such, bind to a defined region of DNA, known as peroxisome proliferator response elements (PPREs), in the promoter region of specific genes to regulate their expression. *Iso*-α-acids seems to be able to induce PPAR-α expression and to act as their agonist. On the other hand, *Iso*-α-acids reduce PPAR-γ expression, although it could work on this receptor as partial agonists.

Four forms of PPARs are described (PPAR-α, PPAR-β, PPAR-γ, and PPAR-δ), each of which is characterized by three functional domains: the ligand-binding domain, the *N*-terminal, and the DNA binding domain. After the ligand binding, PPARs form a heterodimer with the retinoid-X receptor (RXR) and, as such, bind to a defined region of DNA, known as peroxisome proliferator response elements (PPREs), in the promoter region of specific genes to regulate their expression [25]. Examples of PPAR agonists are Thiazolidinediones (TZDs) or Glitazones, like rosiglitazone and pioglitazone with high affinity for PPAR-γ and used to improve insulin sensitivity in type II diabetes, and Fibrates like fenofibrate, which are agonist of PPAR-α and used to treat dyslipidemia. Recently, it has been discovered that IAA from hop are also able to influence the activity of both PPAR-α, involved in lipid metabolism, and PPAR-γ, highly expressed in adipose tissue where it regulates the genes responsible for adipocyte growth, differentiation, and lipid storage [26] (Figure 7).

To determine the activity of hop bitter compounds on PPARs, luciferase reporter assay was performed. An increase in luciferase activities was observed following IAA’s addition to CV-1 cells and HepG2 cells, which were co-transfected with a luciferase reporter plasmid (pG5 luc) and an expression vector for human PPAR-γ and PPAR-α, respectively. *Iso*-humulone, *iso*-cohumulone, and *iso*-adhumulone could increase the luciferase activity in a dose-dependent manner when added to CV-1 (1‒3‒10 μM). Specifically, the concentration of 10 μM has shown the ability to induce luciferase activity of 3.8-, 3.5-, and 2.8-fold, respectively. These activities were comparable to those obtained using pioglitazone (1 μM), a PPAR-γ-selective agonist. *Iso*-humulone and *iso*-cohumulone also increased the luciferase activity in a dose dependent-manner when added to HepG2 cells. It was seen that at the concentration of 10 μM, they stimulated luciferase activity of 3.2 and 1.9-fold, while 30 μM of *iso-*humulone had almost the same activity of 3 μM fenofibrate, a specific agonist of PPAR-α. Moreover, *iso*-adhumulone did not affect the PPAR-α receptor, indicating that, in the activation of PPAR-α receptors, may be involved the side chain of *iso* humulone [26].

Although these data have shown *iso*-α-acids ability in activated PPAR-γ receptors, they seem to act differently upon their expression. Recently, the effect of IAA (as a powdered form of the potassium salts of *iso-*humulone) on PPAR-α and -γ expression has been evaluated on human hepatocytes (PHH) and hepatic stellate cells (HSCs). Incubation of steatotic PHH with IAA appears to reduce the expression of PPAR-γ, which normally was significantly increased in steatotic human hepatocytes, and to increase PPAR-α expression, which plays a role in lipid metabolism leading to a reduction in hepatocyte lipid accumulation. Contrarily, in HSCs, IAA was able to improve the expression of both PPAR-α and -γ in a dose-depended manner (5‒10‒20 μg/mL) [27]; this may be an important result since it has been seen that the activation of PPAR-γ determines anti-fibrotic effects on HSCs.

The impact of IAA on PPARs has also been assessed in vivo. The administration of isomerized hop extract and fenofibrate to C57BL/6N mice significantly increased relative liver weight. This activity may result from PPAR-α activation since hepatomegaly was reported as a consequence of the increased activity of PPAR-α receptors [28]. However, in another study on induced hepatic steatosis mice model, if the intake of IAA (500 mg/kg body weight for 15 weeks) did not induce a significant difference in PPAR-α expression, it significantly reduced the expression of PPAR-γ, a key enzyme for lipid synthesis and storage [27]. The same activity on PPAR-γ expression was observed in adipocytes of C57BL/6J mice treated with a high-fat diet plus 5% isomerized hop extract for 20 weeks since a reduction in adipocyte lipid accumulation was demonstrated [29].

In the light of the above, it can be said that IAA can induce the expression of PPAR-α and act as their agonist while they reduce PPAR-γ expression, although it can work on this receptor as an agonist.

#### 3.3.2. *Iso*-α-acids Reduce the Incidence of Weight Gain

Obesity is characterized by the excessive or abnormal accumulation of adipose tissue, which may interfere with the maintenance of a good state of health. Underlying body weight gain, there is the concept of “*positive energy balance*” by which weight gain results from higher energy intake than energy expenditure [30]. In these conditions, it is possible to witness adipocyte hypertrophy for increased lipid storage, followed by a hypertrophic condition with the consequent increase in the number of adipose cells. It is possible to distinguish two types of adipose depots: visceral depots and subcutaneous depots, where the first is known to be related to an increased risk of developing insulin resistance, cardiovascular disease (CVD), and metabolic syndrome features [31]. Several reports are indicating the ability of isomerized hop compounds to prevent lifestyle-related diseases, including obesity.

A study on Diabetic male KK-A^y^ mice fed for two weeks with a diet containing 0.18% (*w/w*) *iso*-humulone, 0.18% (*w/w*) *iso-*cohumulone, or 0.05% (*w/w*) pioglitazone showed that, although the caloric intake was the same, a significant difference in body weight between all groups of mice was observed. In fact, while the two IAA did not induce an increase in body weight, pioglitazone caused a rise of 10% [26]. Similarly, a decrease in body weight was also seen in C57BL/6NCrj fed with a balanced diet (AIN-76A) containing 1% isomerized hop extract (IHE). In comparison, the administration of AIN-76A diet containing 0.05% fenofibrate did not show this effect [28]. More detailed research works have examined the effect of IHE on high-fat diet-induced obesity in C57BL/6N and KK-A^y^ mice. After 6 weeks of feedings, bodyweight of C57BL/6N mice fed with a high-fat diet (HFD) containing 0.2 or 0.6% IHE was reduced in a dose dependent-manner by 14.1 and 22.0% respectively, compared to mice fed with a high-fat diet alone (control group). Further, the ingestion of a standard diet (AIN93G) containing 0.2 or 0.6% IHE for 5 weeks also determined a reduction of body weight by about 9.9 and 13.1%, respectively, compared to the control group fed with AIN93G diet alone. In particular, the ingestion of IHE prevented the gain in weight of subcutaneous, retroperitoneal, and parametrical adipose tissues and of the kidney in a dose-dependent manner. A reduction in body weight, by 9.7 and 10.9%, was also demonstrated in genetically obese KK-A^y^ mice fed with a high-fat diet containing 0.2 and 1.2% IHE, respectively [32]. This evidence suggests that isomerized hop compounds may directly affect body weight reduction, as also highlighted by the fact that mice fed with the same diet are more prone to a weight gain reduction if they receive the IAA supplementation. A reduction in body weight was confirmed after hop extract administration to ovariectomized mice; indeed, in these animals it was observed protection against visceral adiposity increase and liver triglyceride accumulation [33].

An explanation for these IAA activities could derive from their effects on the expression of enzymes involved in the absorption and storage of lipids in epididymal white adipose tissue (WAT). Another study demonstrated that *iso*-humulones can determine a fair low increase in mRNA levels of PPARγ-regulated adipocyte differentiation-related protein (ADRP) and lipoprotein lipase (LPL) genes. Conversely, treatment with 0.05% *w/w* pioglitazone significantly increased both adipocyte ADRP and LPL by 2- and 1.7-fold, respectively [26]. These results are promising since ADRP is the major component of the membrane covering fat globules in different cell types, such as adipocytes and hepatocytes, where it plays an important role in the storage of triglycerides. Besides, it is a specific pre-adipocytes marker and, in fact, the increase in ADRP mRNA expression is considered the earliest indicator of adipocyte differentiation. On the other hand, LPL hydrolyses the circulating triglycerides by releasing fatty acids absorbed by the adipose cell. Thus, unlike pioglitazone, the IAA reduced adipocyte differentiation and lipid storage. These effects could explain the reduction of adipose tissue weight and adipocyte hyperplasia observed after 20 weeks of administration of 2 or 5% hop extract plus high fat-diet to C57BL/6J mice [29]. Furthermore, these activities may also contribute to the reduction of liver steatosis exhibited after the administration of 500 mg/kg IAA to C57BL/6 mice fed with a high-fat diet since a reduction in adipose tissue expansion and hepatocytes lipid accumulation was observed [27].

Researchers also tested the effect of reduced IAA on weight-gain; particular attention was reserved to tetrahydro *iso*-α-acids (also named META060) activity. C57BL/6J mice fed with an HFD supplemented with 0.1% META060 exhibited a significant decrease in total adiposity compared to HDF mice, as both subcutaneous and visceral adipose depots were significantly decreased [34]. Moreover, META060 appears to work by inhibiting fat accumulation in HDF-fed mice, in fact, at the end of the treatment, mice quickly began to gain weight [35].

Therefore, both IAA and reduced IAA seem to play an important role in preventing body weight; on the contrary, weight gain is observed after pioglitazone administration. Since obesity is one of the typical side effects of PPAR-γ full agonists, it is possible to think that isomerized hop products could act as partial agonists on these receptors. This can be also demonstrated by the weak increase, generated by IAA, of genes expressions regulated by PPAR-γ. Furthermore, it was demonstrated that partial PPAR-γ agonists, like halofenic acid, have displayed a weak adipogenic activity on human adipocyte cell lines, and the absence of body weight increases in obese Zucker (*fa/fa*) rats [36].

#### 3.3.3. Iso-α-acids Effects on Glucose Homeostasis and Insulin Resistance

Obesity is one of the main causes of type 2 diabetes, clinically highlighted by hyperglycemia. The alteration of glucose homeostasis is caused by impaired signal transduction through insulin signaling proteins. As a result, there is a lower glucose intake by muscle tissue, an altered lipogenesis, and an increase in liver glucose production [37]. Several phytochemical compounds have been studied as potential tools to regulate glucose homeostasis; particular attention has been paid to IAA’s hypoglycemic activities [6,38].

A study conducted on obese male C57BL/6N mice fed with a high-fat diet has shown that treatment with IAA can improve insulin resistance and glucose intolerance after 12 weeks. In particular, IAA administration revealed low fasting plasma insulin levels (2396 ± 520 and 1605 ± 570 pg/mL for vehicle-treated and 100 mg *iso-*cohumulone-treated mice, respectively) and an improvement in insulin sensitivity, as demonstrated by the decrease in IR index (2217.3 ± 792.9, 1094.4 ± 259.2, 1034 ± 259.2, and 1240.2 ± 259.5 for vehicle-treated, 10 mg *iso*-cohumulone-treated, 100 mg *iso*-cohumulone-treated, and 100 mg isohumulone-treated mice, respectively). Moreover, after the glucose tolerance test (OGTT), mice treated with *iso-*cohumulone (10 and 100 mg/kg/die) showed a reduction in plasma glucose level than mice treated with a vehicle at all time points. In the same way, after insulin tolerance test (ITT) the administration of *iso*-cohumulone for 10 days showed a higher glucose-lowering effect than in vehicle-treated animals [24]. The ability of IAA in improving glucose homeostasis was also shown after the administration of a high-fat diet containing 0.2 or 0.6% IHE to C57BL/6N mice. In this case, although IHE only showed a tendency to reduce insulin resistance during the ITT test, a significant reduction in plasma glucose levels was found during OGTT [32].

In addition to IAA, the hypoglycemic activity of reduced IAA was also evaluated. Specifically, the effect of 100 mg/kg tetrahydro *iso-*α-acids (also named META060) was compared with that of 1 mg/kg rosiglitazone (PPAR-γ agonist) in C57Bl/6J male mice fed with HFD. Both META060 and rosiglitazone were found to improve glucose homeostasis and prevent HDF-induced insulin resistance in the short and long time. The oral glucose tolerance test, performed during week 5 of the dietary intervention, showed that both META060 and rosiglitazone significantly decreased plasma glucose levels, as demonstrated by the mean AUC, which was 20% and 15% lower than HFD-fed mice (*p* < 0.05), respectively. Moreover, after 14 weeks of dietary intervention, META060 was able to reduce fasting blood glucose and fasting insulin concentration in comparison to the level found in untreated mice (4.5 ± 0.3 versus 5.9 ± 0.3 mmol/L, *p* < 0.05 and 0.14 ± 0.05 versus 0.42 ± 0.09 ng/mL, *p* < 0.001, respectively) [35]. According to these data, another study showed that C57BL/6J mice fed with a high-fat diet supplemented with 0.1% META060 (HFD-META060) for 8 weeks displayed an improvement in glucose homeostasis and the inability to develop insulin resistance [34]. Since the effect of META060 on insulin sensitivity is very similar to that of rosiglitazone, it is possible to think that these hop derivatives could be acted by activating PPAR-γ. However, previously we have observed that, unlike TDZs, IAA, and reduced IAA effectively prevent other effects related to PPAR-γ activation, such as weight gain. Thus, an alternative mechanism can be suggested. In particular, it is possible to speculate that IAAs’ ability to improve glucose homeostasis and insulin sensitivities could be traced back to their partial agonism on PPAR-𝛾 and their agonism on PPAR-*α* receptors. These hypotheses are supported by the evidence that PPAR-γ partial agonists, like pyrazol-5-ylbenzenesulfonamide derivatives, have marked antidiabetic effects [39] and by the ability of fenofibrate, a PPAR-*α* agonist, to normalize hyperinsulinemia and hyperglycemia in obese mice [40].

#### 3.3.4. Iso-α-acids Improve Plasma Lipid Profile

Hypertriglyceridemia is a common lipid abnormality that often manifests itself as a consequence of visceral obesity, metabolic syndrome, and type 2 diabetes [41]. Several pharmacological therapies are currently used to lower the lipid profile and improve lipoprotein metabolism. Fibrates are at the frontline in the treatment of marked hypertriglyceridemia [41]. Still, pioglitazone, a member of the PPAR-γ agonist, has been shown to improve both lipid profile and glucose blood levels when used as monotherapy or in combination with other drugs such as metformin of sulphonylurea [42]. Different *in vivo* investigations have been performed to assess hops’ bitter acid activity on the plasma lipid profile.

The administration of a diet containing 0.18% (*w/w*) *iso*-humulone or 0.18% (*w/w*) *iso*-cohumulone to diabetic male KK-A^y^ mice has been shown to reduce plasma triglycerides (62.6% for *iso*-humulone and 76.4% for *iso*-cohumulone, respectively), and free fatty acid levels (73.1% for *iso*-humulone and 84.8% for *iso-*cohumulone, respectively) compared to mice fed with a standard diet. These data were comparable to those obtained after the assumption of food containing 0.05% (*w/w*) pioglitazone (reduction of 60.5% for triglycerides and 69.9%, for free fatty acid) [26]. In support of these results, another study reported that IHE reduced plasma levels of triglycerides (about 24.2% compared to control group) and non-esterified fatty acid (about 38.4% compared to control group) albeit to a lesser extent than fenofibrate (68.2% and 44.7% respectively) [28]. Further, the high-fat diet containing 1% IHE feeding rats had shown a reduction in blood triglyceride levels coupled with an increase in their feces excretion [32]. Pancreatic triacylglycerol lipase is known to be the main enzyme involved in the hydrolysis of dietary triglycerides in adults [43]. However, data from different *in vitro* investigations about IAA inhibitory effect of pancreatic triacylglycerol lipase are contradictory [29,32]. Thus, it is possible to hypothesize that the increase of triglycerides excretion in feces caused by IHE could be due to its skill in reducing PPAR-γ expression and/or activity. In fact, as recently demonstrated, PPAR-γ transcriptionally up-regulates the gene expression of pancreatic triacylglycerol lipase [43].

IHE, consisting mainly of *iso*-humulones, was also coupled with an atherogenic (high-fat and high-cholesterol) diet to assess better its effect on C57BL/6 mice plasma lipid profile. It was shown that IHE (10 mL IHE/kg diet, equivalent to 3 g *iso*-humulones/kg diet) determined a higher reduction in hepatic triacylglycerol content than fenofibrate (0.5 g fenofibrate/kg diet), a PPAR-α agonist usually used in the treatment of hyperlipidemia, (33% and 7% reductions, respectively). Further, the supplementation of IHE prevented a decrease in HDL-cholesterol at the low dose (2 mL IHE/kg diet, equivalent to 1.1 g *iso*-humulones/kg diet) and significantly raised plasma HDL-cholesterol at the high dose (5 mL IHE/kg diet, equivalent to 2.9 g *iso*-humulones/kg diet, and 10 mL IHE/kg diet, equivalent to 3 g *iso*-humulones/kg diet). These results are excellent, as the administration of an atherogenic diet is known to be coupled with a decrease in plasma HDL-level and a significant increase in the atherogenic index (AI). However, there was no change in the number of LDL particles in mice treated with IHE than mice fed with the only atherogenic diet [44].

**Figure 8:**
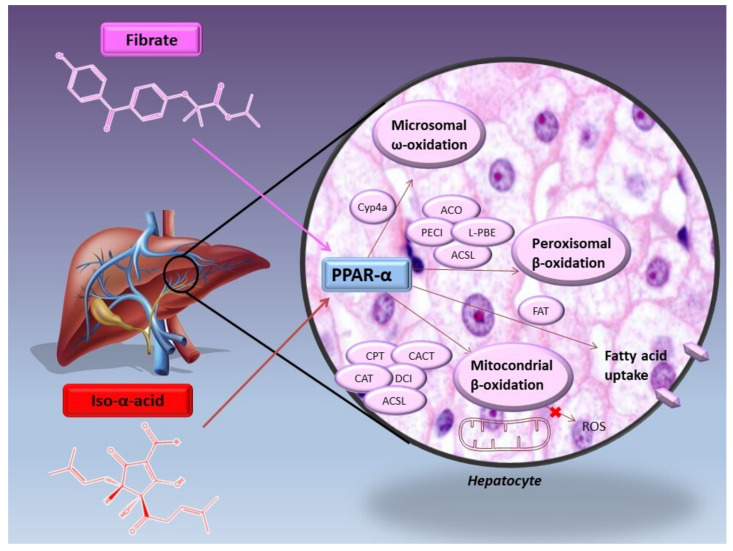
*Iso*-α-acids as well as Fibrate decrease fatty acid and triglycerides plasma levels by up-regulating genes involved in fatty acid up-take, microsomal ω-oxidation, peroxisomal and mitochondrial β-oxidation. These activities exerted by bitter hop compound are related to the activation of PPAR-α with a mechanism very similar to that of fibrates.

To better define the mechanism underlying the ability of IAA in improving lipid profile, a quantitative real-time RT-qPCR analysis of mRNAs genes expression was performed on the liver of mice treated with IHE. Fibrates are known to reduce triglycerides levels through PPAR-α activation, followed by the transcriptional up-regulation of LPL and down-regulation of apolipoprotein CIII (ApoCIII). In the same way as fenofibrate, IHE increased LPL expression, implicated in triglyceride hydrolysis of chylomicrons and VLDL particles, and decreased ApoCIII expression, thereby reducing plasma triglycerides levels. Moreover, as fenofibrate and other PPAR-α agonists, IHE also decreased fatty acid and triglyceride levels by stimulating the expression of genes involved in microsomal ω-oxidation and peroxisomal β-oxidation (Figure 8). In particular, IHE improved the expression of Cyp4a family, like Cyp4a14, 10, and 12, involved in the ω-oxidation of long-chain fatty acids following the release of dicarboxylic acid. IHE also increased peroxisomal β -oxidation pathway and thus the expression of peroxisomal enoyl-CoA hydratase/L-3-hydroxyacyl-CoA dehydrogenase bifunctional enzyme (L-PBE), acyl-CoA oxidase (ACO), and delta3-delta2-enoyl-CoA isomerase (PECI), involved in the degradation of dicarboxylic acids in addition to very long- and straight long-chain fatty acid [28]. In supports of these activities comes the result obtained from a quantitative real-time RT-PCR analysis of mRNAs for ACO and fatty acid translocase/CD36 (FAT) genes performed on the liver of KK-A*^y^*and C57BL/6N mice treated with *iso*-humulone or *iso*-cohumulone. Both mRNA levels of ACO, involved in peroxisomal β-oxidation of fatty acids, and FAT genes, implicated in the uptake of long-chain fatty acids through the cell membrane, were increased by *iso-*humulone or *iso*-cohumulone (ACO mRNA levels by 1.6-, 1.7-fold, and FAT mRNA levels by 2.9- and 3.3-fold, compared to KK-A*^y^* control mice, respectively) [24]. Although to a lesser extent than in microsomal ω-oxidation and peroxisomal β-oxidation, IHE also up-regulated the expression of genes involved in mitochondrial β-oxidation, like carnitine palmitoyl transferase 2 (CPT2), carnitine/acylcarnitine translocase (CACT), carnitine acetyltransferase (CAT), and mitochondrial delta3-delta2-enoyl-CoA isomerase (DCI), involved in the short-, medium-, and long-chain fatty acids degradation. In addition, IHE weakly induced the expression of several long-chain fatty acid CoA ligases, such as ACSL1, 4, and 5, which are required for fatty acids activation in both peroxisomal and mitochondrial β-oxidation. All these effects mediated by IHE were almost completely abolished in PPAR-α-deficient mice; thus, bitter isomers of hop may modulate blood lipid status by activating PPAR-α with a mechanism very similar to those of fibrates [28].

#### 3.3.5. Iso-α-acids Reduce Systemic Inflammation

For centuries, folkloric medicine has used plant-derived compounds for the treatment or prevention of inflammatory disorders but, only in recent years researchers have been directed toward the study of the underlying intracellular mechanism of possible herbal anti-inflammatory activity. In particular, several studies on the potential anti-inflammatory activity of hop bitter acid have been performed.

*In vitro* investigations on L929sA fibroblasts showed that IAA could reduce TNF-induced cytokines mRNA expression at higher concentrations (50–100 μM) without affecting the cell viability. In detail, IAA reduced the expression of genes involved in inflammatory processes such as IL-6, RANTES (Regulated upon Activation, Normal T Cell Expressed and Presumably Secreted), and TNF-α in a dose-dependent manner [45]. The transcription of IL-6, RANTES, and TNF-α is known to be regulated by transcription factors, which in turn can be activated by pro-inflammatory mediators, such as cytokines (TNF and IL-1β), pathogens (LPS), and oxidative stress. Nuclear factor-kB (NF-kB) is part of the transcription factors family known to play a key role in developing inflammation. Normally, NF-kB is retained in the cytosol in an inactive form, but pro-inflammatory mediators may provoke its activation by inducing IkB kinase complex (IKK), which leads to the phosphorylation-induced proteasomal degradation of the inhibitor of NF-kB proteins (IkBs). Thus, free NF-kB is transferred into the nucleus where it binds specific DNA promoters-sequence facilitating its transcription [46,47]. It was seen that IAA inhibited dose-dependently and efficiently the transactivation of the pro-inflammatory transcription factors NF-kB, activator protein-1 (AP-1), and cAMP-response element-binding protein (CREB), involved in increasing IL-6 gene expression [45]. NF-kB has been found to have not only a central role in the regulation of IL-6 expression but also in that of inducible nitric oxide synthase (iNOS) and TNF-α. Research demonstrated that pre-treatments of J774.1 cells with *iso*-humulone (25 μg/mL) not only attenuated the LPS-dependent ~30-fold induction of iNOS mRNA, but also reduce LPS-induced nitrite in media and thus NO levels [48]. It is a good result since an increase of iNOS expression is related to the rise of nitric oxide (NO) synthesis, involved mainly in the pathophysiology of inflammation, as it governs a broad spectrum of processes such as: inflammatory diseases, recruitment and adhesion of leukocyte, ischemia, wound healing, and tumor-induced angiogenesis [49]. However, *iso*-humulones had only a mild effect on TNF-α mRNA expression [48].

**Figure 9:**
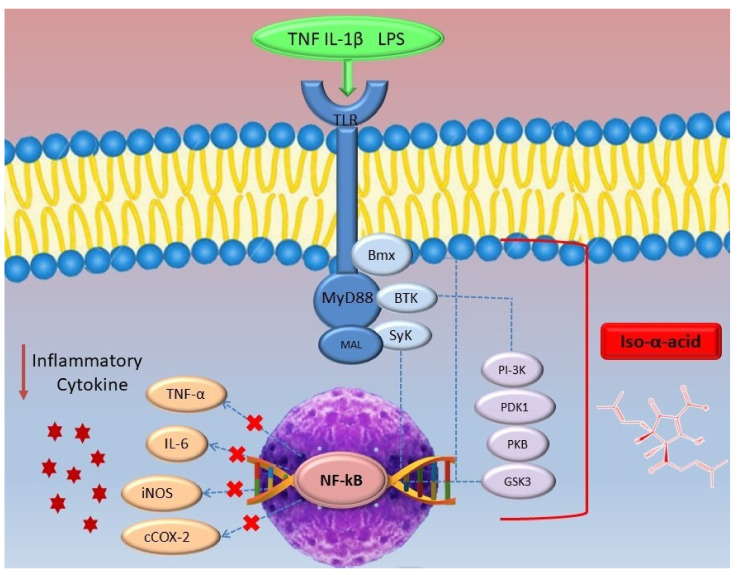
*Iso-*α-acids can exert anti-inflammatory activity by inhibiting the NF-kB pathway by acting on several kinases involved upstream and downstream of the signal transduction. Consequently, the dysregulated secretion of adipokines and pro-inflammatory cytokines related to obesity decreased.

Similar to IAA, reduced *iso*-α-acids, such as RIAA and THIAA, have also been found to inhibit inflammatory signal transduction of NF-kB. In fact, *in vitro* investigations proved that both RIAA and THIAA (1–20 μg/mL) dose-dependently reduced NF-kB nuclear translocation and abundancy in lipopolysaccharide (LPS)-stimulated RAW 264.7 macrophages [50]. This activity was shown to be similar to that of parthenolide, a natural NF-κB inhibitor that works through the inhibition of NF-κB activation and releases from the cytoplasmatic IkB complex [50,51]. Furthermore, RIAA and THIAA inhibited several protein kinases involved in LPS-triggered activation of toll-like receptors (TLRs). These reduced *iso*-α-acids appeared to inhibit BTK and SyK, kinases involved in the phosphorylation of TLR and, consequently, in the initiation of a series of signaling cascades implicated in the activation of MAPKs and NF-kB, leading the production of pro-inflammatory cytokines [50,52]. Particularly, a study conducted on LPS-stimulated RAW 264.7 macrophages demonstrated that THIAA inhibited the activity of BTK (IC_50_ = 41 μg/mL), SyK (IC_50_ = 60 μg/mL), and Bmx (IC_50_ = 87 μg/mL) in up-stream LPS activated signalosome [53], a signalling complex consisting of several proteins, including adapter proteins MyD88, recruited after the activation of TLR [54]. The effect of THIAA on PI3K, PKB, PDK, and GSK3 has also been studied, as these kinases play an important role in the downstream LPS-activated signalosome, leading to the activation of NF-kB. It was seen that THIAA inhibited multiple kinases in the PI3K pathway dose-dependently (1-20 μg/mL), including all three isoforms of PI3K (β IC_50_ = 54 μg/mL; δ IC_50_ = 15 μg/mL; γ IC_50_ = 15 μg/mL), PDK1 (IC_50_ = 53 μg/mL), PKBβ (IC_50_ = 30 μg/mL), and both isoforms of GSK3 (α IC_50_ = 28 μg/mL; β, IC_50_ = 17 μg/mL) [53] (Figure 9). These findings were also confirmed by another *in vitro* study on hepatic stellate cells (HSC) treated with hop bitter acid extract. NF-kB is also involved in the activation of HSC, playing a key role in the development of hepatic steatosis [55]. Thus, reduced *iso*-α-acid inhibits multiples kinases involved upstream and downstream of the signal transduction, leading to NF-kB inactivation.

These results may explain the anti-inflammatory effects of THIAA demonstrated in both TNF-α or LPS-activated human monocytic cell lines (THP-1). In fact, pre-incubation of THP-1 cells with different THIAA concentrations (1–20 μg/mL) reduced the expression of many inflammatory factors, including IL-1β, IL-10, monocyte chemoattractant protein 1 (MCP-1), RANTES, and MIP-1α, in a dose-dependent manner. MCP-1 and RANTES play an important role in the development of atherosclerosis by working in different ways: MCP-1 strengthens the adhesion of monocytes to the vascular-1 adhesion molecules expressed on endothelial cells, promotes their transmigration in the intima, and stimulates their maturation in macrophages; RANTES is a chemokine secreted by platelets able to interact with P-selectin and is involved in the mediation of monocytes/macrophages infiltration in atherosclerotic lesions. The reduced expression of these mediators results in a decreased adhesion of monocytes to endothelial cells, as also demonstrated by the low binding of TNF-α-stimulated THP-1 monocytes to TNF-α activated human aortic endothelial cells (HAECs) after pre-treatment with THIAA [56]. Therefore, by inhibiting transcription factors, such as NF-kB and AP-1, *iso*-α-acid and reduced *iso*-α-acid can improve either inflammation or the first stage of the atherosclerotic process.

Since NF-kB can act upstream of cyclooxygenase 2 (COX-2) by controlling its gene transcription, the effect of reduced IAA on this enzyme has also been evaluated. Cyclooxygenase is involved in the conversion of arachidonic acid into prostaglandin H_2_ (PGH_2_), which in turn is converted by different enzymes into various prostanoids such as prostaglandin E2 (PGE_2_), prostacyclin, and thromboxane A2 (TXA_2_). Two different isoenzymes of COX have been identified: COX-1, a constitutive enzyme, thus always present in the body, and COX-2, which can be constitutive (cCOX-2), at the level of the central nervous system, kidney, and intestine, or inducible (iCOX-2). Pro-inflammatory mediators such as LPS, TNFα, or interleukin-1β (IL1β) can induce COX-2 expression [57]. An *in vitro* investigation on RAW 264.7 macrophages showed that the addition of RIAA and THIAA 1 h prior to LPS stimulation inhibited PGE_2_ formation, presumably through the inhibition of COX-2. In particular, the addition of 20 μg/mL THIAA 1 h before the stimulation of macrophages with LPS exhibited a higher inhibition in COX-2 abundancy than RIAA (reduction of 78% and 60%, respectively) [50]. The same *iso*-humulones ability in inhibiting PGE_2_ production was also confirmed by an *in vivo* investigation on male Fischer 344 rats [58]. Moreover, another study demonstrated that RIAA could inhibit selectively iCOX-2 stimulated PGE_2_ production in RAW 264.7 macrophages. Contrarily, an investigation conducted on AGS gastric mucosal cells showed minimal effect on cCOX-2 and COX-1 as revealed by the low PGE_2_ inhibition by RIAA (IC_50_ = 21μg/mL) compared to that of celecoxib (IC_50_ = 0.024 μg/mL), aspirin (IC_50_ = 0.52 μg/mL), or ibuprofen (IC_50_ = 0.57 μg/mL) [59]. Therefore, basing on these results, it is possible to say that reduced *iso*-α-acids, unlike non-steroidal anti-inflammatory drugs (NSAIDs), could have lower toxicity against the gastrointestinal tract where COX-2s are constitutively expressed.

To date, the nuclear receptor (NR) superfamily has been extensively studied for its anti-inflammatory effect, so researchers have wondered whether the anti-inflammatory effect of IAA may also be due to an action on these factors. Particular attention has been paid to glucocorticoid receptor alpha (GRα), which has been demonstrated to reduce inflammation through the down-regulation of pro-inflammatory mediators and the trans-activation of anti-inflammatory proteins. For example, dexamethasone performs its anti-inflammatory activity by acting on GR. A comparison of dexamethasone (DEX) and IAA activity showed that, while DEX increased the luciferase activity of a GRE-containing reporter by about 12-fold in L929sA cells, IAA, at concentrations which give significant repression of NF-kB activation (50 μM), did not increase the GRE-driven promoter activity [45]. Thus, it has been demonstrated that IAA do not inhibit the transcriptional activity of NF-kB by acting at this level.

IAA and reduced *iso*-α-acid have also been tested *in vivo* to determine their potential therapeutic application in treating inflammation. Studies on Zymosan-induced inflamed paw in female C57BL/6 mice model demonstrated that 250 μg IAA administration reduced paw swelling, a typical symptom of zymosan-induced inflammation. Moreover, following its anti-inflammatory dose-dependent activity demonstrated *in vitro*, the administration of a higher dose of IAA, equal to 1 mg, led to a further reduction of oedema [45]. The anti-inflammatory activity of bitter hop compounds has also been compared with that of celecoxib, an NSAID belonging to the family of selective COX-2 inhibitors. Fourteen days of oral administration of 10, 50, or 250 mg/kg RIAA and THIAA reduced the arthritis index in a dose-dependent manner, and the activity of 250 mg/kg RIAA and THIAA appeared the same as 20 mg/kg of celecoxib. Furthermore, the histological evaluation of joint from sacrificed animals had shown that while celecoxib only reduced swelling, RIAA and THIAA also reduced joint degradation [50]. It is known that joint degradation results from several inflammatory mediators’ activity, many of which are under NF-κB control. This may explain why drugs that only act on COX-2 do not prevent this process, while RIAA and THIAA attenuate inflammation and tissue degradation by acting upstream of the NF-κB pathway. THIAA was also tested on obese and type 2 diabetic mice; the integration of HFD with 0.1% THIAA resulted in an increase of about 46% of IL-10 (HFD 72.7 ± 3.9, HFD+THIAA 106.3 ± 13.2), an anti-inflammatory cytokine able to protect against diet-induced insulin resistance and to improve insulin sensitivity. Moreover, the levels of G-CSF, a key pro-inflammatory regulator of diet-induced obese mice, were normalized by the supplementation of THIAA (CT 53±5.1, HFD 105.7±28, HFD+THIAA 50.6±3.6) [34]. These selective COX1/2 inhibitory properties and the consequent inflammation reduction were also demonstrated after hop extract administration to C57BL/6 mice model of zymosan-induced arthritis [60].

Recent evidence in rodents and humans have shown that elevated gut-derived endotoxins (LPSs) can play an important role in obesity-related disorders [61]; indeed, LPS is closely linked with plasma glucose levels, fasting insulinemia, and type 2 diabetes incidence [62]. It was seen that the addition of THIAA to HFD made mice significantly resistant to HFD induced metabolic endotoxemia. This result could be explained by THIAA’s ability to increase the activity of intestinal alkaline phosphatase (IAP), which is involved in maintaining intestinal integrity and detoxification from LPS. In fact, in mice fed with HFD+THIAA, it was seen an increase of IAP activity by about 25% compared to the HFD group [34]. Several factors, such as cytokines, diet, and diseases, can affect intestinal barrier function and increased permeability with the consequent increase in pathogenic bacteria’s translocation [63]. Thus, the effect of THIAA on the maintenance of intestinal integrity has also been studied. THIAA was shown to reduce gut permeability by modulating occludin and ZO-1 tight-junction proteins in high-fat diet-induced obese and type 2 diabetic mice model. In particular, HFD+THIAA administration to mice resulted in increased ZO-1 and mRNA occludin levels of about 2- to 3-fold, respectively [34]. These data can be explained not only by THIAA’s ability to increase IAP activity but also by its ability to reduce TNF-α and IL-1β levels through the down-regulation of NF-kB [45]. These activities are complemented by the hop alpha acids’ antimicrobial activity by disrupting cellular transmembrane pH gradient. A recent *in vitro* study has indeed demonstrated hop extract’s effect on human gut bacterial consortium composition and metabolism [64].

Therefore, in light of the above, it can be said that both *iso*-α-acids and reduced *iso*-α-acids may reduce the state of chronic low-grade inflammation related to metabolic syndrome by acting on several targets.

### 3.4. Clinical Studies

The healing activities of hops have also been tested on humans. As demonstrated by *in vivo* studies, *iso*-humulones have shown the ability to reduce weight gain in pre-diabetic and diabetic individuals. In particular, in a study conducted on pre-diabetic patients (age: 44–65, fasting glucose levels: 110–125 mg/dL, HbA1c: 5.2–6.4%, Body Mass Index: 24–30), capsules containing *iso*-humulones were administered daily for 12 weeks following different protocols: 4 placebo capsules (group A), 2 test capsules containing 16 mg of *iso*-humulones (group B), 4 test capsules containing 32 mg of *iso*-humulones (group C), or 6 test capsules containing 48 mg of *iso*-humulones (group D). During the 12° week, a significant reduction in body weight and BMI was observed in group D. The waist circumference decreased significantly from the 0-week value after 4 weeks in groups B, C, and D while hip circumference decreased significantly from the 0-week value after 4 weeks in group D (*p* = 0.0062), and after 12 weeks in group B (*p* = 0.0398). At the 12 week it was also evidenced a decreased in total fat area (TFA) [in group C (*p* = 0.0010) and D (*p* < 0.0001)], in visceral fat area (VFA) [in group D (*p* ≤ 0.0001)], and in subcutaneous fat area (SFA) [in group C (*p* = 0.0113) and D (*p* = 0.00010)] [65]. It was also paid particular attention to glucose homeostasis and haemoglobin A1, which indicates the average blood sugar level in the last 2–3 months. It was found that the administration of 100 mg IHE (equivalent to 80 mg of isohumulones) to diabetic patients for 12 weeks led to a significant reduction in blood glucose and haemoglobin A1c levels by 10.1 and 6.4%, respectively [26]. The same reduction of these parameters was shown following the intake of 32 mg of the encapsulated *iso*-humulones [65], indicating that even the administration of fewer doses *iso*-humulones can improve glucose homeostasis.

As demonstrated in a randomized clinical trial, the reduction of weight gain, observed in these studies, can be directly related to reducing hunger as a consequence of the activation of bitter taste receptors after the ingestion of encapsulated bitter hop extract [66]. In the recent year, several studies have demonstrated that the activation of bitter taste receptor expressed into the gastrointestinal tract is directly related to an increased sense of satiation as a consequence of gut peptides release like peptide YY (PYY) and glucagon-like peptide 1 (GLP-1) [9,67]. In particular, the hop compound’s bitter taste was found to be mediated by three bitter taste receptors, TAS2R1, TAS2R14, and TAS2R40. Thanks to structural bioinformatics analysis it was found that these receptors can recognize hop-derived-compound through a conserved asparagine in transmembrane 3. Meanwhile, chemoinformatics analysis revealed that hop bitter acids cluster in another region of the receptors’ chemical space when compared to other bitter compounds. This indicates that hop bitter compounds may constitute a new class of bitter compounds [68].

The activity of KDT501, the potassium salt of the *n*-(isobutyl) congener of a tetrahydro *iso*-α-acid, has also been tested. Contrary to *iso*-humulones, the administration of 300, 600, or 800 mg KDT501 twice daily for 7 days to insulin-resistant patients did not lead to a significant reduction in body weight, nor were changes in glucose homeostasis, in the levels of haemoglobin A1c, and insulin sensitivity. However, there was an improvement in lipid metabolism, as demonstrated by a significant decrease in post-meal triglycerides [69]. Previously, it was seen that *iso*-humulones could reduce plasma triglyceride levels by increasing LPL expression in mice liver. Since KDT501 is structurally similar to *iso-*humulones, it is possible to think that the decreased post-meal triglycerides could be for increased liver lipoprotein lipase activity, which leads to increased clearance of triglyceride-rich lipoprotein. Furthermore, KDT501 treatment displayed increased total and high-molecular-weight adiponectin and a significant decrease in TNFα [69]. Adiponectin is an anti-inflammatory adipokine expressed and secreted only by adipose tissue, so an increase in this mediator indicates an improvement of adipose tissue function. An adiponectin gene expression analysis showed that KDT501 increased adiponectin secretion by a post-transcriptional mechanism in subcutaneous WAT taken by biopsy. Moreover, it was observed that KDT501 could control the expression of genes involved in the control of adipose tissue lipid synthesis, uptake, and storage. In particular, KDT501 administration to obese, non-diabetic, and insulin-resistant subjects resulted in a reduction of the mRNA expression of ACACA (0.86-fold; *p* = 0.038), involved in regulating fatty acids synthesis and DGAT (0.87-fold; *p* = 0.043), which regulate triglyceride formation [70]. These results confirm previous data obtained *in vivo* and *in vitro*. In fact, *in vitro* investigations have demonstrated indeed that KDT501 acted as a partial agonist of PPARγ and that exerted an anti-inflammatory activity on monocytes/macrophages. Furthermore, the administration of KDT501 to diet-induced obesity mice and diabetic fatty rats reduced body fat, fed glucose level, glucose/insulin AUC following an oral glucose bolus, plasma haemoglobin A1C, total cholesterol, and triglycerides [71].

Regarding the anti-inflammatory activity of reduced IAA, an ex vivo evaluation was performed on peripheral blood mononuclear cells (PBMC) isolated from a healthy patient after a single oral administration of 940 mg THIAA. It was found that the isolated and washed PBMC were refractory to LPS-stimulated IL-6 and TNF-α production, indicating that THIAA gave PBMCs anti-inflammatory activities that make them refractory to LPS stimulation. Therefore, blood THIAA concentration, which was found between 5 and 15 μg/mL, could suppress systemic inflammation [53]. Similarly, plasma collected from subjects treated with 600 mg RIAA showed anti-inflammatory activity when applied to RAW 264.7 cells, followed by LPS stimulation, as demonstrated by the inhibition of PGE_2_ biosynthesis. It was also found that the 6-week administration of 500 mg RIAA b.i.d. to subjects with knee osteoarthritis resulted in a significant reduction of the WOMAC osteoarthritis index and of the VAS score used as a pain assessment. Because of RIAA’s potential anti-inflammatory effect, the potential clinical gastrointestinal toxicity was also tested using fecal calprotectin, a biomarker of gastrointestinal mucosal inflammation. This marker was measured at baseline, 7, and 14 days after dosing with 450 mg RIAA b.i.d. or 500 mg naproxen b.i.d. It was seen that while naproxen increased faecal calprotectin by about 241% and 154% over baseline in the first and second weeks, respectively, RIAA did not show an increase in fecal calprotectin [59]. Therefore, RIAA’s anti-inflammatory effect is associated with an absence of gastrointestinal toxicity, which is known to be a typical side effect of NSAIDs. However, NSAIDs are known to be not only toxic to the gastrointestinal tract but also to the cardiovascular system, as demonstrated, for example, by the lengthened platelet closure shown after aspirin intake. Thus, potential toxicity of RIAA on cardiovascular homeostasis was assessed using the urinary excretion of PGI-M and TXB_2_, two metabolic products of PGI_2_ and TXA_2_ known as a potent vasodilator and vasoconstrictor, respectively. It was seen that while the daily administration of 400 mg celecoxib to 8 subjects who had never used NSAID in the last week decreased the rate of PGI-M excretion by 19% (*p* < 0.05) without effecting TxB_2_, the intake of RIAA did not affect the excretion of these prostanoids [72]. These results confirm previous *in vivo* investigations where RIAA administration increased the ratio of PGI-M and TXB_2_ to 1.07 (95%CI = 1.38‒0.80), while the administration of celecoxib decreased this ratio to 0.71 (95% CL = 1.04‒0.44) [59]. The effect obtained with the use of celecoxib is linked to the inhibition of both iCOX-2 and cCOX-2 with a consequent reduction in prostacyclin synthesis (PGI_2_), an arachidonic acid product with vasodilatory and anticoagulant effects. In this condition, there is a disruption of the balance between thromboxane (TXA_2_), an eicosanoid produced by COX-1 of platelet, and prostacyclin, thus increasing atherosclerosis, thrombogenesis, and the risk of cardiovascular complications [73]. Therefore, the results obtained from clinical investigations support *in vivo* data: reduced *iso*-α-acids can reduce only the activity of iCOX-2s with minimal effect on cCOX-2s and COX-1s.

Finally, about the IAA effect on cardiovascular parameters, only a study had reported a reduction in systolic blood pressure after the assumption of capsules containing 100 mg of IHE (equivalent to about 80mg of isohumulones) twice daily for 12 weeks [26]. Moreover, a randomized crossover clinical trial demonstrated that the ingestion of IHE exerts beneficial effects on endothelial function by increasing the flow-mediated vasodilatation (FMDs). This effect is probably determined by reducing intracellular oxidative stress, as demonstrated by *in vitro* experiment on human aortic endothelial cells (HAECs) [12]. In contrast, other clinical investigation showed no significant changes in systolic or diastolic blood pressure [59,72]. Thus, further clinical investigations should be carried out on the potential anti-hypertensive activity of IAA and reduced *iso*-α-acid.

## 4. Material and Methods

### 4.1. Search Strategy

Following the Preferred Reporting Items for Systematic Reviews and Meta-Analyses (PRISMA) guidelines, the systematic search of the literature was performed in April 2020 and included all reports published to December 2020. It included the article inherent to the review’s object found on two specialized databases: Scopus and PubMed. Full-text not available were not requested and unpublished data was not pursued further. The keyword used to launch the articles were: *Humulus lupulus*, bitter acid, *iso*-humulone, and *iso*-α-acid, paired with the following words: metabolic syndrome, obesity, inflammation, diabetes, pharmacokinetics, clinical trial.

### 4.2. Study Selection

The inclusion criteria for the manuscript writing included pre-clinical studies (*in vivo* and *in vitro*) and clinical studies involving the use of *iso*-α-acid, reduced *iso*-α-acid, and isomerized bitter hop extract. Only articles in English and with the keyword in the title or abstract were selected. Other review articles, retrospective studies, meta-analysis, abstracts, letters, editorials, and manuscripts without full text available were not considered for the formulation of this systematic review. For selecting the articles, three independent investigators (M.P.; D.R.; F.L.) first selected the document according to the title and abstract and then analyzed full-texts. In cases of dissensus, other independent reviewers were consulted (L.M. and I.F.). All selected articles were carefully reviewed to include or exclude manuscripts that did not agree with the criteria described. 

### 4.3. Data Extraction

All the selected articles were carefully analyzed and information regarding bitter hop acid activity, as well as study design, experimental models, doses used, major outcomes, and general mechanism of action, were extracted. The articles containing the most important results were synthesized in two tables (Table 2 and Table 3).

### 4.4. Methodological Quality Assessment

The authors assessed the quality of each investigation and the risk of bias by adapting the checklist of *Cochrane Handbook for Systematic Reviews of Interventions*, adjusted explicitly for animal intervention study (SYRCLE’s) [74] and clinical trials [75]. The evaluation of the studies’ qualities was done basing on the presence or absence of the information reported in Table 4 and Table 5. Studies that did not follow all the criteria were linked as articles with a medium risk of bias, while manuscript characterized by a lack of these criteria were included in the high risk of bias group. Finally, investigations that respected all parameters were assessed as having a low risk of bias.

## 5. Conclusions

The hydrophilic IAA from bitter hop acids, as well as their reduced derivatives, have been proven to be effective molecules able to influence several biological targets commonly used for medical nutrition therapies. Bitter compounds were able to positively influence metabolic syndrome-related diseases when tested *in vitro* and *in vivo*, from animal models till clinical studies, thanks to their pleiotropic influences. In several cell lines, IAA have demonstrated to act as agonists on PPAR receptors and as anti-inflammatory molecules by inhibiting multiples kinases involved in NF-kB activation. Moreover, their effectiveness was also tested on animal models. In fact, these bitter molecules have been shown to have a beneficial effect on weight gain, lipid metabolism, glucose homeostasis, insulin sensitivities, and inflammation by acting on different targets. They have also been shown to reduce systemic endotoxemia and improve gastrointestinal mucosal integrity in HFD mice models. Furthermore, IAA appear to be safe phytochemical compounds able to reduce inflammation without toxic impact on the cardiovascular system and gastrointestinal tract commonly affected by NSAIDs. Similarly, they mitigate insulin resistance and hyperglycemia without typical TDZs side effects such as weight gain or oedema. The impact on human health was assessed confirming the significant reduction of glycemia and related parameters. All these aspects make IAA excellent candidates to treat metabolic syndrome disorders, although further studies should be done to assess its toxicological profile.

## Figures and Tables

**Table 1:** To predict the transport of molecules across cells membranes it is necessary to know their maximum calculated diameter (dmax/nm) and they calculated partition coefficient (ClogP). Blanco et al. demonstrated that isomerized *H. lupulus* compounds have a molecular length not exceeding 1.5 nm indicating a great ability to spread through the cell membrane. Further, it was demonstrated that isomerized α-acids and their reduced derivatives have a good grade of lipophilia, as indicated by their ClogP value [19].

Compound	dmax/nm	Clog P
*Iso*-cohumulone	1.1664	2.90
*Iso*-humulone	1.3620	2.90
*Iso*-adhumulone	1.3830	2.90
Dihydroisohumulone	1.3536	2.80
Tetrahydroisohumulone	1.3851	3.00
Hexahydroisohumulone	1.3609	3.70

**Table 2:** Description of *in vivo/in vitro* mechanisms involved in the decreased incidence of metabolic disorders.

Compound	Model	Treatment Time	Doses/Concentration	Biological Activity	Reference
*Iso-*humulone *Iso-*cohumulone *Iso-*adhumulone	CV-1 cells		*Iso-*humulone/*iso-*cohumulone/*iso-*adhumulone 1‒3‒10 μM	↑PPAR- γ	[24,26]
	HepG2 cells		*Iso-*humulone/*iso-*cohumulone/*iso-*adhumulone 3‒10‒30 μM	↑PPAR-α	
	KK-A^y^ male mice	2 weeks	HFD + 0.18% (*w/w*) *iso-*humulone/*iso-*cohumulone	↓plasma triglycerides ↓free fatty acid ↑glucose tolerance ↓fasting plasma insulin levels ↓IR index ↓body weight ↑liver ACO gene expression ↑liver FAT gene expression ↓white adipose tissue ADRP gene expression ↓white adipose tissue LPLgene expression	
	C57BL/6N female mice	10 weeks 14 days	HFD + 0.18% (*w/w*) *iso-*humulone/*iso-*cohumulone HFD +10–100 mg/kg/day *iso-*humulone/*iso-*cohumulone		
IHE (*iso*-humulone, *iso*-cohumulone, and *iso*-adhumulone)	C57BL/6NCrj mice PPARα^-/-^/129S4/SvJae mice	1 week	AIN76A + 1% IHE/AIN76A + 2% Chol + 1% IHE	↓plasma triglycerides ↓nonesterified free fatty acid ↑PPAR-α ↑microsomal ω-oxidation ↑peroxisomal β-oxidation ↑mitochondrialβ-oxidation	[28]
IHE (*iso*-humulone, *iso*-cohumulone, and *iso*-adhumulone)	HepG2 cells		90 μg/mL	↑PPAR-α	[32]
	C57BL/6N female mice	6 weeks 5 weeks	HFD + 0.2–0.6% IHE AIN93G standard diet + 0.2–0.6% IHE/HFD + 0.2–0.6% IHE	↓body weight ↓plasma triglycerides ↑glucose tolerance ↑faeces lipid content ↓pancreatic triacylglycerol lipase activity ↑liver ACO gene expression ↑liver MCAD gene expression ↓ liver DGAT2 gene expression	
	KK-Ay male mice	5 weeks	HFD + 0.2–0.6% IHE		
	Wistar rat		HFD + 1% IHE		
HE	Cloned mouse 3T3-L1 fibroblasts C57BL/6J male mice C57BL/6 ovariectomized female mice	20 weeks 12 weeks	0–500 µ/mL HE HFD + 2 or 5% HE 400 mg/kg HE	↑PPAR- γ expression ↓body weight ↓liver weight ↓mesenteric adipose tissue weight ↓epididymal adipose tissue weight ↓PPAR-γ expression ↓liver TC ↓liver lipids (TC and TAG) ↓plasma lipids (TC and TAG) ↓plasma glucose levels	[29,33]
IHE1 (humulones, lupulones and *iso*-humulones) IHE1 (*iso*-humulones) Purified *iso*-humelones	C57BL/6NCrj female mice	Experiment1 1 week 2 week Experiment2 1 week Experiment3 2 week Experiment4 1 week	Atherogenic diet Atherogenic diet + 2 mL IHE1/kg Atherogenic diet + 5 mL IHE1/kg Atherogenic diet + 10 mL IHE2/kg Basal diet Basal diet + 10 mL IHE2/kg Cholesterol diet + 10 mL IHE2/kg Cholesterol diet Cholesterol diet + 0.5 g Fenofibrate Cholesterol diet + 10 mL IHE2/kg Cholesterol diet Cholesterol diet + 10 mL IHE2/kg Cholesterol diet + 3 g purified *iso*-humulones	↓body weight ↑HDL-cholesterol ↓plasma triacylglycerol levels ↑ACO mRNA levels ↑ACS mRNA levels ↑LPL mRNA levels ↑FATP mRNA levels ↑HMGS mRNA levels ↑LDLR mRNA levels ↓Apo AI mRNA levels ↓Apo CIII mRNA levels	[44]
*Iso*-humulone	PHH cells HSC cells		5–10–20 μg/mL	↑PPAR-α ↑PPAR-γ expression in HSCs cellsγ expression in PHH cells ↓SCD1 expression ↓FASN expression ↑CPT1 expression ↑ACOX1 expression	[27]
	C57BL/6N mice	15 weeks	WTD + 0.5%(*w/w*) *iso*-humulone	↓weight gain↓adipose tissue expansion ↓leptin ↑adiponectin ↓insulin resistance γ expression ↑mitochondrial β-oxidation ↓HMOX1 expression ↓SCD1 expression ↓FASN expression ↓JNK pathway	
THIAA	C57BL/6J mice	8 weeks	HFD + 0.1% THIAA	↓body weight ↓total adiposity ↓subcutaneous and visceral adipose depots ↑glucose homeostasis ↓hyperinsulinemia ↑IAP activity ↑ZO-1 mRNA levels ↑occludin m-RNA levels	[34]
THIAA	C57Bl/6J male mice	14 weeks	HFD + 100 mg/kg THIAA	↓ weight gain ↓total body fat ↑glucose tolerance ↓fasting insulin levels	[35]
KDT501	THP-1 cells 3T3L1 murine preadipocytes Human subcutaneous adipocytes C57Bl/6J male mice ZDF male rats	30 days 32 days	6.25, 12.5, 25, 50 µM 3.13, 6.25, 12.5, 25 µM 10 µM 25, 50, 100 and 200 mg/kg KDT501 100, 150, or 200 mg/kg KDT501	γ↓MCP-1 ↓RANTES ↓IL-6 ↓fat mass ↓glucose AUC ↓cholesterol ↓thriglycerides	[71]
THIAA	RAW264.7 macrophages		1–5–10–20 µg/mL	↓PEG_2_ production ↓COX-2 protein abundance ↓NO formation ↓NF-kB nuclear abundance and activity ↓BKT (IC_50_ =41 µg/mL) ↓SyK (IC_50_ = 60 µg/mL) ↓BMX (IC_50_ = 87 µg/mL) ↓PI3K (β, IC_50_ = 54 µg/mL; δ and –γ, IC_50_ = 15 µg/mL) ↓PDK1 (IC_50_ = 53 µg/mL) ↓PKBβ (IC_50_ = 87 µg/mL) ↓GSK3(α, IC_50_ = 28 µg/mL; β, IC_50_ = 17 µg/mL)	[53]
*Iso*-humulone	L929sA mouse fibrosarcoma cells		50–100 μM	↓IL6 protein expression ↓TNF-α protein expression ↓RANTES protein expression ↓NF-kB-dependent gene expression ↓AP-1-driven gene expression ↓CREB-driven gene expression	[45]
RIAA and THIAA	RAW264.7macrophages		1–2.5–5–10–20 µg/mL	↓NF-kB nuclear abundance and activity ↓PEG_2_ production ↓COX-2 protein abundance ↓TNF-α production ↓iNOS expression ↓NO production ↓BKT ↓SyK	[50]
	DBA/1J female mice	14 days	10–50–250 mg/kg	↓arthritis index ↓joint degradation	
RIAA	RAW 264.7 macrophages		1–5–10–20 µg/mL	↓PEG_2_ production ↓iCOX-2 protein abundance	[59]
THIAA	THP-1 cells HAEC cells		1–5–10–20 µg/mL	↓IL6 expression ↓TNF-α expression ↓RANTES expression ↓MCP-1 expression ↓MMP-9 expression ↓NF-kB activation ↓AP-1 activation ↓monocyte-endothelial cell interaction	[56]
IAA-rich extract	J774 A.1 murine macrophages		25 mg/mL	↓iNOS expression ↓NO production ↓IL6 expression ↓TNF-α expression	[48]
	C57Bl/6 J female mice	4 days	0.75 mg/kg	↓plasma triglycerides ↓iNOS expression	
IHE *Iso*-humulones	RAW264.7 cells Male Fischer rats	2 weeks	2.5, 5 µg/mL *iso*-humulones 10, 25 µg/mL IHE AIN-76A + 0.01%, 0.04%, 0.05% IHE	↓PEG_2_ production	[57,58]

Abbreviations: Isomerized hop extract IHE; Hop extract HE; *iso-*α-acids IAA; Rho-*iso*-α-acids RIAA; Tetrahydro-*is*o-α-acids THIAA; Potassium salt of the n-(isobutyl) congener of a tetrahydro *iso*-α-acid KDT501; High-fat diet HFD; Western type diet WTD.

**Table 3:** Description of the principal clinical effects of hop isomerized compounds.

Compound	Clinical Investigation		Biological Effect	Ref.
	Dose	Time of Treatment		
*Iso*-humulone	16–32–48 mg 80mg	12 weeks 8 weeks	↓body weight ↓total fat area ↓BMI ↓fasting blood glucose ↓HbA1c ↓systolic blood pressure ↓GPT, GOT, γGTP blood levels	[24,26,65]
IHE	Capsules containing 10.8 mgIHE/10 kg body weight	30 and 120 min after ingestion	↓triglyceride levels ↓glucose levels ↓insulin levels ↑FDM ↑NO ratio	[12]
Bitter hop extract	100 or 250 mg	24 h	↓hunger reduction	[66]
KDT501	escalating doses from 200 to a maximum dose of 1000 mg every 12 h	From 7 days to 28 days	↓total cholesterol ↓post-meal triglycerides ↑adiponectin ↓TNF-α ↓ACACA mRNA expression ↓DGAT mRNA expression	[69] [70]
THIAA	940 mg	Single oral dose	↓systematic inflammation ↓TNF-α ↓IL-6	[53]
RIAA	600 mg 500 mg 450 mg	Single oral dose 6 weeks 14 days	↓iCOX-2 ↓PGE_2_ ↓WOMAC Global score ↓VAS score ↓CYP2C9 (IC_50_ = 0,25 µg/mL) ↓CYP2C19 (IC_50_ = 6.1 µg/mL) ↑PGI-M/TXB_2_ ratio	[59,72]

Abbreviations: Rho-*iso*-α-acids RIAA; Tetrahydro-*iso*-α-acids THIAA; Potassium salt of the n-(isobutyl) congener of a tetrahydro *iso*-α-acid KDT501.

**Table 4:** Checklist for assessment of risks of bias in pre-clinical studies [75].

Checklist for Assessment of Risks of Bias in Pre-clinical Studies
Are the hypothesis and objective of the study clearly described?
Are the main outcomes to be measured clearly described?
Are the main findings of the study clearly described?
Are the samples size calculations reported?
Are the animals randomly housed during the experiment?
Are the investigators blinded from knowledge which treatment used?
Are the outcome assessors blinded?
Is the dose/route of administration of the HCQ properly reported?
Is the dose/route of administration of the drug in co-treatment properly reported?
Is the frequency of treatments adequately described?

**Table 5:** Checklist for assessment of risks of bias in clinical studies [75].

Checklist for Assessment of Risks of Bias in Clinical Studies
Are the hypothesis and objective of the study clearly described?
Are the main outcomes to be measured clearly described?
Are the main findings of the study clearly described?
Are the samples size calculations reported?
Are the animals randomly housed during the experiment?
Are the investigators blinded from knowledge which treatment used?
Are the outcome assessors blinded?
Is the dose/route of administration of the HCQ properly reported?
Is the dose/route of administration of the drug in co-treatment properly reported?
Is the frequency of treatments adequately described?

## Data Availability

Not applicable.

## Abbreviation

ADME	Absorption, distribution, metabolism, and excretion
AP-1	Activator protein-1
ACO	Acyl-coa oxidase
ApoCIII	Apolipoprotein CIII
PPREs	As peroxisome proliferator response elements
AI	Atherogenic index
CREB	cAMP-response element-binding protein
CAT	Carnitine acetyltransferase
CPT2	Carnitine palmitoyltransferase 2
CACT	Carnitine/acylcarnitine translocase
COX-2)	Cyclooxygenase 2
PECI	Delta3-delta2-enoyl-coa isomerase
DEX	Dexamethasone
DHIAA	Dihydro-*iso*-α-acids
FAT	Fatty acid translocase/CD36
GLP-1	Glucagon-like peptide 1
GRα	Glucocorticoid receptor alpha
OGTT	Glucose tolerance test
HSCs	Hepatic stellate cells
HIAA):	Hexahydro-*iso*-α-acids
HDL	High-density lipoprotein
HFD	High-fat diet
HAECs	Human aortic endothelial cells
PHH	Human hepatocytes
IKK	IkB kinase complex
VFA)	Visceral fat area
iNOS	Inducible nitric oxide synthase
IkBs	Inhibitor of NF-kb proteins
ITT	Insulin tolerance test
IL-6	Interleukin-6
IL1β)	Interleukin-1β
IAP	Intestinal alkaline phosphatase
IHE	Isomerized hop extract
IAA	*Iso*-α-acids
LPS	Lipopolysaccharide
LPL	Lipoprotein lipase
LDL	Low-density lipoprotein
MetS	Metabolic Syndrom
DCI	Mitochondrial delta3-delta2-enoyl-coa isomerase
MCP-1	Monocyte chemoattractant protein-1
NO	Nitric oxide
NSAIDs	Non-steroidal anti-inflammatory drugs
NF-kB	Nuclear factor-kb
PYY	Peptide YY
PBMC	Peripheral blood mononuclear cells
L-PBE	Peroxisomal enoyl-coa hydratase/L-3-hydroxyacyl-coa dehydrogenase bifunctional enzyme
PPARs	Peroxisome proliferator-activated receptors
ADRP	PPARγ-regulated adipocyte differentiation-related protein
PGE2	Prostaglandin E2
PGH2	Prostaglandin H2
RANTES	Regulated upon Activation, Normal T Cell Expressed and Presumably Secreted
RXR	Retinoid-X receptor
SFA	Subcutaneous fat area
THIAA	Tetrahydro-*iso*-α-acids
TZDs	Thiazolidinediones
TXA2	Thromboxane A2
TLRs	Toll-like receptors
TFA)	Total fat area
TNF-α	Tumor necrosis factor-α
WAT	White adipose tissue
